# The violence of reproductive injustice: Reflections on birth control and its medical epistemics

**DOI:** 10.1186/s12939-025-02727-5

**Published:** 2025-12-08

**Authors:** Jana Niemann, Lisa Glaum, Dennis Jepsen, Lea Hofmann, Liane Schenk, Amand Führer

**Affiliations:** 1 Institute of Medical Sociology (IMS), Interdisciplinary Center for Health Sciences, Medical School of the Martin Luther University Halle-Wittenberg, Halle, Germany; 2 Corporate Member of Freie, Institute of Medical Sociology and Rehabilitation Science, Charité – Universitätsmedizin Berlin, Universität Berlin and Humboldt-Universität zu Berlin, Berlin, Germany; 3 Institute for Medical Epidemiology, Biometrics, and Informatics (IMEBI), Interdisciplinary Center for Health Sciences, Medical School of the Martin Luther University Halle-Wittenberg, Halle, Germany

**Keywords:** Hormonal contraception, Contraceptive counseling, Patient-provider interaction, Gynecological violence, Reproductive justice, Structural violence

## Abstract

**Background:**

The widespread promotion of oral contraceptives raises concerns about side effects, informed choices, and contraceptive coercion, which contribute to gynecological violence influenced by systemic factors. The link between gynecological violence and oral contraceptives is understudied and rarely examined through systemic violence theories.

**Methods:**

To address this gap, we explored how a continuum of symbolic, structural, and slow violence manifests in the physical body by drawing on qualitative online interviews with 19 former oral contraceptive users and six gynecologists in Germany. Data were analyzed using reflexive thematic analysis informed by symbolic, structural, and slow violence theories. This process involved iterative coding, theme development, and discussions within the research team.

**Results:**

Using the conceptual framework of gynecological violence, we show how the dominance of medical and pharmaceutical knowledge, systemic neglect of contraceptive counseling, and prioritization of oral contraceptives over other methods of contraception contribute to a cycle of symbolic and structural violence, ultimately harming users through slow violence.

**Conclusion:**

To promote more equitable and inclusive contraceptive counseling, we recommend advancing gender-responsive research, expanding the rights-based and psychosocial counseling offered by different health professionals (and not just physicians), and enhancing gynecology training programs to better prepare gynecologists for contraceptive counseling. Ultimately, these measures aim to transform contraceptive care into a more equitable, informed, and patient-centered practice.

**Supplementary Information:**

The online version contains supplementary material available at 10.1186/s12939-025-02727-5.

## Introduction

The ability of an individual to control reproduction is a fundamental human right that is vital for sexual and reproductive health [1]. Access to modern contraception is essential to exercising this right. However, access alone is insufficient, and individuals must also have agency to choose a contraceptive method. Recognition as a human right means valuing individual needs in contraceptive decision making [2]. Therefore, contraceptive counseling should empower patients to make informed decisions about their contraceptive choices, whether it involves selecting a new method, discontinuing the current one, or switching to an alternative option [3]. However, the practical implementation of these rights in healthcare settings often falls short; contraceptive care is shaped by coercion [4, 5], misinformation [6], or dismissal of patients’ concerns [7, 8].

These challenges are particularly evident in contraceptive pills. Although biomedical discourse often highlights the effectiveness and safety of hormonal contraception [9], more critical research underscores the experience of side effects [10–12], the dismissal of such side effects by healthcare providers [5, 7], pressure on contraceptive choices [13], and inadequate information about the potential risks of contraceptive pills and non-hormonal alternatives [14, 15]. In addition, the long-term effects of contraceptive pills, such as increased risk of venous thromboembolism [16, 17] and breast cancer [18], have been increasingly studied in epidemiological studies. These concerns highlight broader issues in reproductive health care, particularly the power dynamics that influence medical interactions.

Inadequate counseling and pressure from health care providers can be framed as issues of gynecological violence, a concept recently introduced in a report requested by the European Parliament’s Committee on Women’s Rights and Gender Equality [19]. In this report, gynecological violence is understood to be a harmful practice perpetrated during gynecological care throughout reproductive life. It results from structural and organizational factors that enable and sustain patterns of violent behavior in healthcare settings. While acknowledging that consequences are often physical or psychological, the authors of the report emphasize that the concept also encompasses systemic and institutional practices that perpetuate harm. They further highlight the role of biomedicalization, i.e. the authority of healthcare providers over reproductive and sexual health decisions, and emphasize that “structural issues of the healthcare system [act] as a breeding ground for violence.”. Thus, the report moves beyond an exclusive focus on interpersonal abuse. In this sense, the physical, verbal, and coercive forms of violence documented in the EU report can be situated on a continuum with symbolic violence (Bourdieu [20]), structural violence (Galtung [21]), and slow violence (Nixon [22]), which are enabled and legitimized by broader systemic arrangements, while their consequences often unfold gradually through long-term physical and psychological harm. However, the report defines gynecological violence together with obstetric violence, and much of the cited evidence is derived from research on coercive and violent practices in birth and obstetric care, while other forms of violence in the context of gynecology receive much less attention. As gynecological violence is a new conceptualization, little empirical research supports it, and its theoretical foundations remain underdeveloped.

Crucially, while the authors of the EU report acknowledges that structural and organizational factors enable violent behavior, they treat them as context rather than violence itself. We argue that these conditions are not merely background conditions, but are themselves constitutive of gynecological violence.

In this study, we pursued three interrelated aims:

1. To expand the concept of gynecological violence beyond behavioral acts to include structural and organizational harms in reproductive healthcare.
2. To apply the framework to a new domain: contraceptive care, where symbolic, structural, and slow violence shape decisions and autonomy.
3. To provide empirical evidence that grounds the concept in lived experience—showing how routine clinical practices can constitute violence.

Currently, research on contraceptive pills using the theoretical concept of violence is limited. Despite its relevance, gynecological violence has not been systematically examined using established theories of systemic and structural forms of violence. Our research addresses these gaps using a continuum of symbolic, structural, and slow violence to analyze qualitative interviews with 19 former contraceptive pill users and six gynecologists in Germany. Thereby wee seek to answer the following research question: How is symbolic, structural, and slow violence produced and experienced, and how does this continuum of violence shape (oral) contraceptive method choice?

### Conceptual framework: symbolic, structural, and slow violence and contraceptive counselling

Violence in sexual and reproductive health research is often studied in its visible and interpersonal forms, such as birth control sabotage [23, 24], obstetric violence [19, 25], or sexual abuse [26] that are forms of interpersonal gender-based violence. However, other forms of violence are less obvious, yet they enable, sustain, and exacerbate harm through systemic and institutional means. To theorize gynecological violence as a multidimensional phenomenon, we introduce a continuum of three interconnected forms: symbolic, structural, and slow violence. These forms share an often-invisible nature, embedded in societal norms and institutions, perpetuating harm through indirect or gradual means, disproportionately affecting vulnerable populations.

The first form, *symbolic violence*, according to Bourdieu, is a subtle form of domination that operates through communication and cognition, where dominant ideas are internalized as “natural”, thereby perpetuating oppression [20]. It works through the unconscious internalization of social structures and power relations, shaping everyday habits and perceptions [20, 27]. In contraception, this is closely linked to biomedicalization, which extends medical authority into everyday life through risk management and technoscience [28]. The existing literature argues that pregnancy prevention has been biomedicalized and framed as an individual responsibility for women, emphasizing risk management of pregnancy and contraceptive use [29]. Symbolic violence in contraceptive pill use operates by withholding information, silencing women’s concerns, and maintaining unequal doctor-patient power dynamics, as Roso [30] demonstrates. A key mechanism of this is the hierarchical construction of knowledge, where patients’ vernacular knowledge [31], shaped by personal experience and social networks [32–34], is systemically devalued as biomedical research and providers prioritize clinical expertise and control [9, 31, 34–37]. Research shows that providers often prioritize statistically significant risks (such as thrombosis) over the lived experiences with side effects that impact quality of life, effectively dismissing patients’ lived realities [38]. Furthermore, symbolic violence unfolds its effects through the gendered construction of contraceptive responsibility, with women bearing the burden of pregnancy prevention through contraceptive pills [39, 40]. This gendered hierarchy normalizes the control of women’s reproductive choices, constituting a form of violence [41, 42].

The second form, *structural violence*, introduced by Galtung [21] and expanded by Farmer [43, 44], describes how societal systems and institutions (differentially) harm individuals and populations. It is ‘structural’ because the underlying mechanisms are deeply embedded in society’s political and economic fabric and ‘violent’ because they produces injury, often among those least responsible for inequalities [44, 45]. Although its effects on bodies may be visible, the underlying mechanisms remain obscure. While no study has directly applied this concept to contraception, scholars have identified the structural mechanisms of power that restrict reproductive choice. These include systemic forces such as heterosexism [46], racial [47] and economic [33] inequalities, and neoliberal forces [29, 48] that shape contraceptive-decision-making. Economic barriers, such as out-of-pocket costs and lack of insurance coverage, are recognized as significant structural impediments to contraceptive access and choice [33]. Furthermore, the healthcare system perpetuates structural harm through the systemic neglect of safe, and less-invasive long-term contraceptive options for all sexes [49] and the lack of research on post-discontinuation [50]. These structural forces restrict sexual and reproductive choices and normalize harm within routine care, perpetuating gynecological violence.

The third concept, *slow violence* defines harm that “occurs gradually and out of sight, [it is] a violence of delayed destruction that is dispersed across time and space” [22]. Initially developed to analyze gradual environmental damage [22, 51], the concept can be applied to the contraceptive pill, where long-term side effects emerge over time [10, 52, 53] often leading to discontinuation [12, 54, 55]. Misinformation [6, 56], uncertainty regarding unwanted effects [36], and the systemic failure to provide transparent, comprehensive information create a cumulative erosion of reproductive autonomy, representing invisible, attritional harm that exemplifies slow violence.

Within this continuum, the act of discontinuing a method can be understood not merely as a medical choice but as a form of strategic norm negotiation [57]. Rather than outright opposition, individuals may strategically “play social norms off each other” [57], for instance, by mobilizing the increasingly prevalent ideal of the “natural” body [57] to counteract the medical expectation to use hormonal contraception [55, 57]. This act disrupts the power dynamics that condition patients to be compliant and can be seen as a form of resistance against the cumulative slow violence of medical neglect and the symbolic violence inherent in the dismissal of their experiences.

With this marked gap in current research, we selected theories that could address this gap because they were distinct from other perspectives on violence, particularly interpersonal violence. This aligns with research arguing that a narrow focus on individual determinants ignores the larger structural systems that shape violence against women [58]. Rather than framing gynecologists as deliberate perpetrators or patients as passive victims, our approach acknowledges the complexity of clinical interactions by focusing on the structural and systemic dimensions of power that shape these encounters. This perspective is crucial because different forms of violence —structural, symbolic, and interpersonal—are not mutually exclusive but are deeply interrelated [22, 58, 59].

Drawing on multiple theoretical traditions, we aim to capture the complex ways in which violence in gynecological care is enacted, legitimized, and sustained. Integrating these different theories strengthens the critical analysis of qualitative data [60, 61] and has been applied previously in sexual and reproductive health research. Such theoretical integration has been applied previously in sexual and reproductive health research. For instance, Hourani et al. [41] and Tarpey-Brown et al. [42] examined gendered forms of structural and symbolic violence and their connections to interpersonal violence among migrant women. Similarly, Jung [25] showed how symbolic, structural, and institutional forms of violence intersect in the context of birth. These studies highlight the importance of incorporating multiple theoretical lenses, as they highlight how violence operates across different levels simultaneously. Furthermore, integrating slow violence alongside symbolic and structural violence broadens the analytical scope. While structural violence exposes hidden harm to social systems, it assumes a static structure. By contrast, slow violence captures how harm becomes invisible through temporal delay, only emerging as its effects accumulate [22]. By combining these perspectives, we can analyze not only how violence is embedded in structures (structural) and legitimized through norms and authority (symbolic), but also how it unfolds cumulatively and often imperceptibly (slow). This integration of theoretical perspectives therefore provides a more comprehensive lens for understanding the continuum of gynecological violence.

Following Scheper-Hughes’ and Bourgois’ [59] proposition, we view these forms of violence as one end of a *continuum*. In the context of gynecological violence, direct interpersonal violence (e.g., verbal abuse, coercion, or non-consensual procedures) often coexists with the more hidden forms, which intersect and reinforce each other [27], embedding violence into both practice and structure. Moreover, gynecological violence is a feminist concern operating within the framework of gender, (hetero-)sexism, medicalization, and misogyny [4, 25, 29, 46], which is fundamentally structural and political [19].

We propose that this continuum of gynecological violence, combining symbolic, structural, and slow violence, is embedded in the different discriminatory trajectories of power structures and inequities [25] and mediates provider-patient micropolitics [62] in counseling counselling. A key aspect of this is the hierarchical construction of knowledge [62], where providers decide (1) what information to share, 2) how to frame the risks and benefits [36], and 3) which side effects are considered real [38], often leaving contraceptive seekers with only their embodied vernacular contraceptive knowledge [31] before, during, and after use as a reference point [39].

**Fig. 1:**
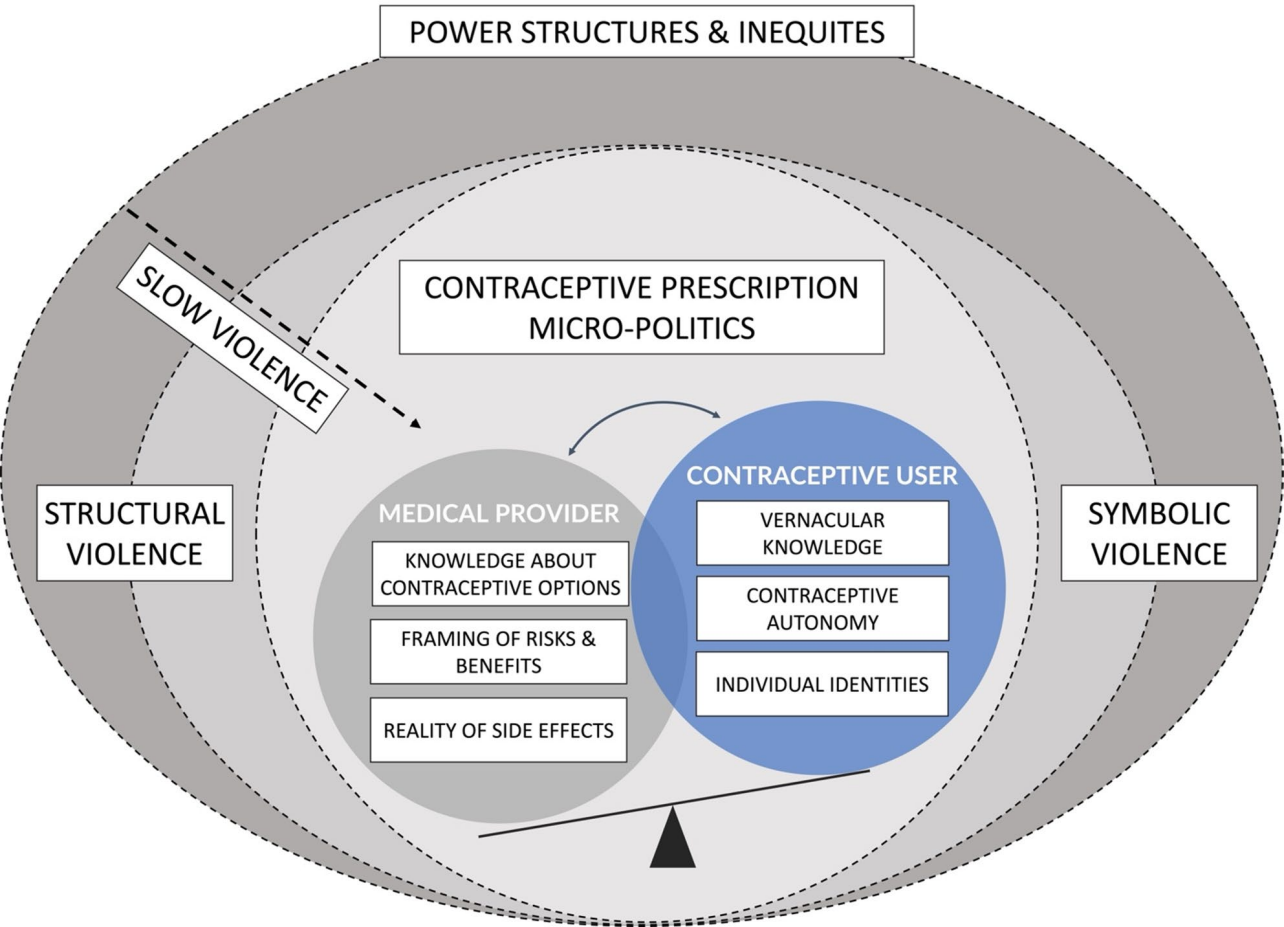
The symbolic, structural, and slow violence in contraceptive prescription micro-politics

Figure 1 depicts how symbolic, structural, and slow violence mediates contraceptive prescription politics. The outer circle symbolizes discriminating power structures and inequities, such as classism, sexism, and racism, which impact all forms of violence [25]. The second circle is the continuum of symbolic [20], structural [44], and slow violence [22]. The inner circle depicts contraceptive prescription micropolitics [62], which are influenced by the asymmetric knowledge and power relationship between the medical provider and contraceptive user. This opens up the possibility of interpersonal contraceptive coercion/violence [4].

## Study context

Contraceptive pills require prescriptions from outpatient gynecologists [63]. Physicians in statutory health systems are legally required to make cost-effective decisions [64]. Reimbursed contraception access depends on age and circumstances; otherwise, the costs are out-of-pocket [65]. Contraception decision-making has historically been neglected in German gynecology, with clinical guidelines only recently published [66, 67]. For the past ten years, information on hormonal contraception risks has been primarily disseminated through official letters that warn physicians about emerging evidence for previously unknown side effects of drugs. Although gynecologists are the primary source of contraceptive information [12, 68, 69], users often seek alternative sources, such as the internet [12]. This may lead to disconnection between providers [12]. In particular, consultations about contraceptive pills are influenced by (hetero-)sexist gender norms in gynecological practice [46]. Additionally, preferences for hormonal contraception [70] exist in the gynecological field.

## Methodology

We did not use our theoretical framework to inform the data collection and generation process. We conducted an inductive, reflexive thematic analysis [71, 72] to examine the manifestations and consequences of violence within this framework. Our analysis is grounded in critical realist ontology, which “assumes an ultimate reality but claims that the way reality is experienced and interpreted is shaped by culture, language, and political interests” [73]. This approach allowed us to generate key themes while contextualizing the interviewees’ experiences within the concept of contraceptive violence. We have situated experiences with broader social and cultural meanings. Using constructionist epistemology, we viewed knowledge as contextually situated, utilizing researcher subjectivity and perspectives as tools for knowledge generation [72]. Latent meanings were generated from the interview data.

### Recruitment and participants

JN conducted online interviews, recruiting former pill users through social media, networks, gynecologists via email, LinkedIn, and a professional organization. Participants received the study information and consent forms, and interviews were scheduled after confirmation of eligibility. Data collection continued until thematic saturation was reached. Although 300 gynecologists were contacted, six participated, sufficient due of content redundancy.

Former contraceptive pill users were predominantly cisgender (*n* = 18, 95%) and heterosexual (*n* = 17, 84%). The median age was 27 years, with most patients (*n* = 13; 68%) between 25 and 34 years. Educational backgrounds included secondary school certificates (*n* = 5; 26%), subject-related higher education qualifications (*n* = 1; 5%), general higher education qualifications (*n* = 4; 21%), bachelor’s degree (*n* = 6; 31.58%), and master’s degree (*n* = 3; 16%). Most patients had multiple contraceptive pill initiations (*n* = 11, 58%).

The gynecologists were aged 47–66 years (mean = 58years), with 13–30 years (mean = 20 years) of practice experience. Four were cisgender females, and two were cisgender males.

### Data generation

We investigated the experiences of former contraceptive pill users (*N* = 19) and gynecologists’ values and behaviors during contraceptive counseling (*N* = 6) in Germany. All interviews were conducted online. JN conducted episodic interviews^1^ with former contraceptive pill users between December 2022 and September 2023, transcribed verbatim. JN and LG conducted semi-structured interviews with gynecologists from February 2024 to June 2024 using noScribe for transcription. Episodic interviews^1^ with former users covered contraceptive pill initiation and discontinuation, experiences after discontinuation, contraceptive counseling, and current methods. This analysis focused on counseling experiences and side effects. Semi-structure interviews with gynecologists included contraceptive counseling practices, assessment of patient knowledge, views on pill (dis)continuation, and understanding of medicalization. The study protocol [74] was published before data collection.

### Data analysis

In line with the view that the purpose of qualitative research is to understand the different meanings that people construct about the world [75], we began our analysis with the aim of constructing semantic meaning [71] that reflected the participants’ views. JN and LG familiarized themselves with transcripts and coded data using MAXQDA (2023–2024), generating patterns related to the research topic. They met weekly after coding the interviews to discuss and refine the codes. JN sorted the codes into the initial semantic themes examined by the research team. Semantic themes were discussed in the qualitative colloquiums of the medical faculty. JN developed these themes into latent ones and created thematic maps. The research team reviewed the definitions of the themes, leading JN to define the final themes.

### Reflexivity

The author team views contraception as a fundamental right to sexual and reproductive health. The author team viewed contraception as a fundamental right to sexual and reproductive health. The first author led this project from conception through completion, with experience in qualitative research and background in public health and medical sociology. As a sexual and reproductive rights activist who discontinued contraceptive pills, she documented her reflections throughout her first reflexive thematic analysis project, through journaling. These reflections were part of weekly meetings with LG and were also incorporated into the discussion within the qualitative research colloquium of the medical school of our university.

### Ethics

This study complied with the ethical guidelines set forth in the latest version of the Declaration of Helsinki. This study was approved by the Ethical Review Board at the Medical Faculty at Martin Luther University Halle-Wittenberg (Reg-Nr. 2021-034). All study participants gave informed consent.

## Results

We constructed six key analytic themes: (1) side effects as normalized harmful trade-offs; (2) contraceptive knowledge as a site of power; (3) the hidden costs of contraceptive care; (4) the contraceptive pill as the default, a disconnect between lived and medical knowledge; (5) hidden costs of misinformation; and (6) breaking free: method discontinuation as an act of resistance. By analyzing the participants’ narratives through the lens of gynecological violence, we identify these themes as manifestations of symbolic, structural, and slow violence, each reflecting distinct dimensions of gendered bodily harm and systemic disregard within healthcare and social contexts (cf. Supplementary Table 1).

### Side effects as normalized harmful trade-offs

Our analysis of the participants’ narratives, especially those of former users, suggests a persistent tension between the medical system’s emphasis on the contraceptive pill as the optimal contraceptive method and the lived experiences of those who report profound biopsychological disruptions.

The former contraceptive pill users in our study expressed experiencing side effects, including “*mood changes*”, “*weight fluctuations*”, and “*altered sexual desire*”, which affect their quality of life. These issues are frequently minimized by their gynecologists who trivialize their impact or depict them as normative female experiences. This normalization is not neutral; it reflects a gendered medical gaze that silences women’s experiences and reproduces structural inequity under the guise of clinical routines. For instance, Heather^2^ expressed disappointment in how her concerns were addressed:

I would have liked more understanding and sensitivity to side effects, risks, and, well, about taking the pill. (…) So, it was always very [concentrated? ] in the direction of ‘From a medical point of view, the pill is still the best thing there is.’ This ignores the social debate regarding the side effects and risks of pills.

Heather’s plea for “*more understanding and sensitivity*” is not just about providing information; it demands epistemic justice. Her frustration with the monologic framing of the pill by the medical system as “*the best thing there is*” reveals a deeper rupture: the erasure of lived experiences and the stifling of a broader social debate about the risks and side effects. These experiences were shared by other former pill users in our study as well. Thereby recognizing the slow impact on their bodies, revealing the systemic failure to provide transparent information about the side effects. As Natalia describes:

I also think that all these side effects, which are listed in the package leaflet alone, should be addressed, and that perhaps other methods, such as this symptothermal method, should be mentioned. If I had not stopped taking the pill, I would not have known of it. And why would I? You do not just google ‘taking your temperature during your cycle,’ do you? And you do not learn it from biology class or gynecologists.

Her statement highlights a systemic silence: non-pharmaceutical methods, such as the symptothermal method, are rarely discussed in clinical or educational settings, meaning that contraceptive seekers are unaware of the alternatives available to them. This absence is not accidental, but rather, through our theoretical framework, is rooted in structural violence, a system that privileges pharmaceutical solutions and marginalizes non-medical, body-based approaches. This is perpetuated by symbolic violence, such as the cultural framing of the pill as the default, rational, and ‘best’ choice. Together, these forms of violence manifest as slow violence: an invisible, cumulative erosion of reproductive autonomy, where a failure to inform can cause prolonged harm, shaping choices, silencing bodies, and side effects over time. In this context, a lack of knowledge is not merely an oversight but a harmful omission. This reveals how the body becomes a site of negotiation between individual agency and societal pressure in the context of reproductive health.

The gynecologist involved in our study explained that while some of their patients associate health issues with the pill, the “*causal relationship is often unclear*” (Dr. Günther). However, they also acknowledged possible side effects to some extent. For instance Dr. Pete, shared:

A significant proportion report that they feel changed in their soul, that they feel sadder, feel slowed down, somehow do not feel themselves, but somehow feel different.

This statement prompted us to critically examine how the medical system often downplays or medicalizes subjective experiences of distress. We interpret Dr. Pete’s comments as a recognition of the emotional and existential dimensions of contraceptive use, which are frequently excluded from biomedical narratives.

### Contraceptive knowledge as a site of power

Gynecologists in our study described the systemic issues that reflect broader patterns of inequality. A key issue is the scarcity of studies on male contraception compared with years of research on contraception as a female responsibility. For instance, Dr Elsa’s statement draws attention to the long-standing imbalance in reproductive health research:

It is really bad that there are still no reversible contraceptives available for men. This has annoyed me since I began my studies. The problem is not that it is so much more complicated for men, but that not so much money is being invested in research.

Her long-standing frustration, rooted in her professional experience, highlights a systemic imbalance in reproductive health research. This critique reveals how economic interests—not medical needs —shape scientific investments. By framing the lack of male contraceptives as a matter of profit, not possibility, her words expose how gendered responsibility is sustained through policy rather than biology. This normalization of contraception as a female responsibility can thereby be viewed as symbolic violence, indicating that societal expectations continue to reinforce traditional gender-based roles in reproductive planning.

This symbolic framing is reinforced by the structural neglect of contraceptive counseling in specialist training (German: *Facharztausbildung*), as discussed by the gynecologists in our study. Despite its relevance to everyday practice, it is absent from the formal curriculum. This educational gap deprives future gynecologists of critical competencies in reproductive health and undermines informed patient-centered care.

Dr. Freya: And so, the biggest problem I see is that it is not an issue in gynecological training. We conducted a 6-year specialist training program. It does not even come up with. You do not have to take a course on it. You do not have to do any further training. Since we are not obliged to complete part of specialist training in outpatient practice, you usually come into practice as a specialist and have no idea about it. […] In this respect, it should be a compulsory part of specialist training.

Dr Freya states that contraceptive side effects are not taught, assessed, or required in gynecological training. She notes that trainees “*do not have to take a course on it*” and often enter practice without outpatient experience. This statement highlights a systemic failure in gynecological training: the exclusion of contraceptive care from formal medical education. By failing to prioritize contraceptive counseling, the medical system sidelines patient autonomy and reinforces provider dominance in reproductive decision making. This educational gap is not accidental; it reflects institutional logic, which is not patient-centered, and the consequences of biomedicalization are not critically examined. The institutionalized transfer of knowledge is a mechanism for reproducing structurally entrenched violence.

This structural gap is further compounded by the pharmaceutical influence on clinical knowledge, as Dr. Elsa noted:

At other training courses, there is sometimes a lecture on contraception in special situations or when new pills come out. Of course, we get our information mainly from the pharmaceutical speakers.

Dr. Elsa’s observation highlights the reliance on industry-funded training, which is often taken for granted. Her use of ‘of course’ emphasizes how normalized this is in her professional culture. This reliance reflects a structural dependency: medical education is shaped by pharmaceutical interests that prioritize profit-driven products. This creates a systemic bias that shapes doctors’ knowledge and patients’ choices, demonstrating how commercial interests influence clinical practice from within. This dynamic constitutes structural violence through the systemic exclusion of viable alternatives, as well as symbolic violence in the form of a knowledge hierarchy that devalues self-knowledge and non-drug methods as legitimate forms of care.

Together, these patterns reveal a cumulative, slow violence that stems not from a single act but from the repeated normalization of ignorance, exclusion of alternatives, and prioritization of profit over care. This harm is delayed, dispersed, and often invisible; however, it gradually erodes reproductive autonomy.

### The hidden costs of contraceptive care

Gynecologists shared that contraceptive counseling or “*speaking with patients in this normal health insurance medicine”* (Dr. Frankie) is generally not well remunerated. By undervaluing thorough counseling, the healthcare system economically disincentivizes time-intensive, personalized patient education and informed decision-making. Doctors face economic pressure to cross-finance these services or must charge out-of-pocket fees.

Dr. Pete echoes this structural issue: the “*standard 20-minute appointment*”, which he deems “*often insufficient*” for meaningful contraceptive counselling. To address this issue, he explained: “*So*,* at some point*,* I said that I would charge a small fee for consultation so that I could take my time. And the women respond positively to this*.” This suggests that they value additional time and attention. The undervaluation of counselling time on a systemic level function as a form of structural violence: it shapes clinical practice by making personalized care financially unsustainable, thereby limiting access to information and support. These structural (economic) conditions are not merely administrative oversights but also reflect deeper power dynamics in the healthcare systems. The system also establishes what constitutes ‘valid’. This is symbolic violence: the normalization of a hierarchy in which pharmaceutical prescriptions are viewed as rational, efficient, and authoritative, while patient education is dismissed as ‘extra’, ‘optional’ or ‘not worth the time’. Dr. Pete’s own words, *‘And the women respond positively to this*”, show how this symbolic order is reproduced: patients recognize the value of time, which is made possible through their own payments and not through institutional support. This solution inadvertently reproduces inequity, as access becomes dependent on financial resources. This structural condition disproportionately affects patients with low incomes, who may be unable to afford even a modest fee, effectively making time-intensive care a privilege rather than a right.

### The contraceptive pill as the default: a disconnect between lived and medical knowledge

Former users consistently felt that contraceptive pills were prioritized throughout their implementation, particularly during the first initiation. As Lola expressed, “So the decision was pretty clear, and the gynecologist did not introduce me to any other contraceptive method. It was more like: ”Ah, this is your first time at the gynecologist.” Then, they talked to me, and then I got the pill, and then the issue was over, so to speak.”. This brief, superficial consultation reflects symbolic violence: the limited discussion on side effects, risks, or alternatives neglects long-term health outcomes. It also underscores the dominance of medical authority over patient agency, in which patients’ voices are subordinated to institutional norms.

When patients’ concerns are dismissed, they feel unsupported and misunderstood, worsening the harm inflicted by the health care system. Heather summarized this by saying: “I always ended up with doctors who were absolute pill advocates, right? Every doctor always told me: “[…] It is my decision, but from their point of view, there are no medical reasons to stop taking the pill.” That was the message from everyone, was it not? They said, “If you’re fine with it, if you tolerate it well, there are no reasons to stop taking it”. Heather’s account highlights a pattern reflecting deeper epistemic dismissal and symbolic erasure of vernacular knowledge: the providers’ ‘listening’ becomes a performance that ultimately upholds the pill as the ‘safe’ choice, framing patient autonomy as compliance rather than genuine informed decision-making. This was echoed by Lily: “[…] I had a gynecologist who was very enthusiastic about the pill. […] Therefore, she did not respond to me at all. And that was always a bit of a setback, where I thought to myself, “okay, I don’t have to philosophize with her […] anymore”, and I just thought it was a shame, because I wanted to stop taking the pill myself and she was actually the driving force behind why I actually continued to take it for four more years.”. The lack of genuine dialogue and the assumption that the pill is the default contraceptive solution without considering individual needs further perpetuate one-size-fits-all approaches in healthcare. Symbolic violence is evident in the way the pill is normalized as the default option, while alternatives are presented as unusual, risky, or inconvenient. This is achieved through language, tone, and institutional framing.

Structural violence is deeply embedded in the systematic barriers that prevent access to alternatives. Although all gynecologists that we interviewed emphasize that they want the woman to “*find what suits her best”* (Dr. Frankie) or that “*contraception advice can only lead to an individual solution*” (Dr. Pete). This principle is consistently constrained in adolescents. A clear exception is made for young patients, especially those under 18 years of age, whose reproductive decisions are frequently viewed as needing professional guidance when making reproductive decisions. As Dr. Elsa exemplifies: “*Nevertheless*,* I still think that a 17-year-old who has never had a gynecological examination and may have had sex five times in her life*,* whether it is really a good idea to insert an intrauterine device for the very first time*,* I am always a bit skeptical about that*.” This pattern is echoed in Dr. Pete’s account: “*I will tell a 15-year-old who has never been to the gynaecologist about* intrauterine devices. *Then I will look her in the eye and say*,* ‘I’m sorry*,* but I can’t imagine being the first person to put anything in there and hurt you. That can’t be how you’re introduced to sexuality. It’s also one reason why girls often start taking the pill right away. What’s the alternative?*”. This reflects structural violence: by failing to provide young people with accurate, accessible information, the system sustains their vulnerability not through inherent naivety but through the deliberate withholding of knowledge, keeping them in a state of epistemic marginalization despite their capacity for informed agency. Providers’ reluctance to insert intrauterine devices in adolescents does not stem from clinical evidence, but from subjective fears of causing discomfort or emotional harm, as evidenced by Dr Pete’s comments: *‘I can’t imagine being the first person to put anything in there*.’ This reflects a paternalistic view of young people as vulnerable, positioning the provider as a gatekeeper. The rhetorical question *‘What’s the alternative*?’ presents the pill as the only acceptable option, thereby closing off discussion of safe and effective long-acting methods. Despite claims of personalised care, this results in a standardised default option of the pill. This is consistent with the experiences of former users, who emphasize the standardized prescription of contraceptive pills during their youth. Medical practices restrict contraceptive freedom by favoring the providers’ preferences over the patients’ potential preferences, thus limiting access to alternatives such as intrauterine devices and implants, and citing anatomical issues and discomfort.

Dr Henry’s statement “*I would not want to campaign against safe contraception at all*,* because I find caring for a teenage pregnancy more stressful*,* if that is not wanted*,* than giving good contraceptive advice.”* reveals how pregnancy is not only framed as a medical risk, but also as an emotional and professional burden. The focus is shifting from patient autonomy to provider burden. This risk perception justifies the pill as the default option, thereby reinforcing a standardized approach. By prioritizing the avoidance of pregnancy over individual choice, the stress experienced by the provider becomes an underlying driver of care, subtly undermining reproductive agency and reinforcing the medicalization of young people’s sexuality.

Failure to prioritize the continuity of care during contraceptive decisions undermines contraceptive autonomy. This is evident in Helena’s experience, where her concerns about the potential psychological effects of hormonal contraception and her consideration of sterilization were met not with exploration or support but with dismissal and diversion. Helena recounts:

I told her that I would like to stop taking the pill precisely because I am not sure whether it has anything to do with the psyche, and then she had already dismissed the idea that this is very unlikely if it was not such a problem before. And I think I had also, yes, I had already mentioned at the appointment that I was thinking about sterilization. She found that even less attractive I think (laughing). She was then very quick: “Yes, why not the partner?” Whether something else would be an option. And (…) then it was clear to me, okay, then I do not want to discuss it any further with the doctor, but if I do, then I will do it a bit on my own. I just felt that I was not getting much support from her in that direction.

This moment highlights how structural and symbolic violence operate in tandem to undermine contraceptive autonomy. Her concerns about the possible psychological effects of the pill were dismissed on the basis that any symptoms must be unrelated if they only emerged after she started taking the pill, prioritizing biomedical explanations over her own embodied experience. This reflects epistemic injustice and grounded in an underlying symbolic order. When she expressed interest in sterilization, the healthcare professional quickly suggested that her partner take responsibility without exploring her motivations or offering alternatives. Although this response could be framed as promoting shared responsibility, it actually deflected the conversation, minimized the patient’s agency, and reinforced the medical default of hormonal contraception. Suggesting that their partner take responsibility without addressing the structural barriers to male contraceptive uptake highlighted how reproductive decisions remain gendered, even when presented as collaborative. Structural violence operates through institutional inertia: For Helena, the lack of support and validation caused her to disengage from the provider and pursue her goals independently. Her experience highlights that autonomy is not just about having options but also about being heard, respected, and supported in making one’s own reproductive choices, even when they challenge the medical norm.

In contrast, Dr. Freya emphasizes the importance of empowering women to make confident and informed choices, suggesting that reclaiming reproductive agency requires both systemic change and a cultural shift toward greater self-trust and awareness. This challenges the structural dominance of hormonal contraception and the symbolic framing of women as passive.

Dr. Freya: I would like to see more courage to encourage women who have confidence and want to do it to use condoms and natural contraception. (…) Safe and good contraception is possible. This also makes you more aware of your own body.

Furthermore, we found conflicting views on biomedicalization and contraceptive pills among gynecologists. They reflect both liberation and control, highlighting concerns regarding medical dependence and pathology. Within this duality, the pill is often the default option without intensively considering alternatives or individual needs, especially for adolescents. This finding reinforces gendered control of reproductive health. In addition, the widespread use of contraceptive pills depends on medical interventions, which are not neutral but are embedded in structural and symbolic violence.

Dr. Pete: I always say, sorry, of course, nothing about this is natural. It would be natural if you had a seventh child. However, contraception is not natural. (…) And so, I take the wind out of the sails in this respect. However, I have never asked this question [about the link of biomedicalization and the pill]. I think I am too dominant, perhaps too masculine, perhaps too well-known in the area for being very upfront with my opinions.”

Dr. Pete’s reflection illustrates how medical authorities can silence questioning and reinforce the contraceptive pill as an unchallenged norm. Simultaneously, Dr. Pete’s portrayal of contraception as ‘unnatural’ involves symbolic violence: it moralizes reproductive control, presents medical expertise as beyond question, and silences patient agency by portraying bodily autonomy as unnatural. His reluctance to question the status quo further entrenches this hierarchy, given his authority over the matter. In contrast, Dr. Marry emphasized the significant impact of contraceptive pills on the body:

Dr. Marry: It is a possibility because it is a drug that intervenes massively in the body. […] And I think we, as doctors, have to be very open about that. […]

Dr. Marry’s statement, “we have to be very open about that”, suggests that full transparency about the bodily impact of hormonal contraception is not yet standard practice, but rather a deliberate, exceptional stance. This linguistic hesitation reveals an underlying norm: that such openness is not routine, but a corrective gesture, highlighting how biomedical authority still shapes what counts as legitimate knowledge and what must be ‘framed’ as exceptional, even when it should be standard.

### The hidden costs of misinformation

Gynecologists in our study expressed reproductive and sexual education gaps among their patients. They emphasize that limited sexual education and understanding of female biology, especially the hormonal system, leaves many ill-prepared for informed contraceptive decisions. This knowledge deficit incurs significant, frequently concealed costs reflecting a broader pattern of structural violence. As Dr Mary observed:

And that in practice, you can hardly make up for it [the missing education] because that simply does not achieve anything. […] It just quickly becomes a case of, take the pill, do not forget to take it, then you will not get pregnant, and not everyone wants to know how it works, and it is unlikely to happen, so someone [the woman] would probably have to ask how it works.

In her quote, Dr. Mary highlights that reducing contraception to a mechanical routine — “take the pill, do not forget” – constitutes symbolic violence, normalizing passive compliance over informed agency. Without knowledge of hormonal biology or how methods work, women must navigate complex systems with limited insight, often bearing the burden of seeking answers they may not even realize they need to seek. This knowledge gap has tangible equity implications, including an increased risk of unintended pregnancies, discontinuation of medication due to unexplained side effects, and diminished bodily autonomy. This cumulative, invisible harm reflects the slow violence of systemic neglect. Failure to provide comprehensive and accessible reproductive education creates a systemic barrier that disproportionately affects marginalized populations. True reproductive justice cannot be achieved without equitable access to accurate information, open dialogue, and the right to knowledge.

Inconsistent clinical messaging about hormonal contraception led to uncertainty, especially when treatment was stopped. As former user Hannah described:

You hear a lot about it [the discontinuation], and I think I do not know enough about it because I often hear from my gynecologist that it is a miracle cure-all and there is nothing wrong with it. It is just a few hormones, and there is nothing wrong with them. (…) But then you hear from my friend’s gynecologist “no, there are many better things, and you should not pump your body full of these hormones.

The absence of coherent guidance left many feeling insecure about stopping the pill, particularly when healthcare professionals dismissed their concerns or provided minimal explanations. Former users reported turning to the Internet to understand symptoms, withdrawal effects, and alternatives, highlighting a critical gap in patient-centered care. Without reliable and accessible information from healthcare providers, former users were left to navigate complex decisions alone, often relying on unverified sources. This process not only marginalizes their agency but also subjects them to disciplinary discourses that label their self-sought knowledge as unreliable, thus reproducing hierarchical power imbalances.

In addition, some gynecologists in our study criticized the existence of economic barriers, especially for low-income individuals, in accessing contraception. Incomplete coverage creates inequities, limiting marginalized groups’ healthcare access and autonomy. As Dr. Freya stated: “*I would like to see the funding for contraceptives. Perhaps it should be limited to those who have little money. […] I would like to see free access to them. [contraception*]”. This call for targeted, publicly funded access highlights that financial barriers undermine the reproductive autonomy of marginalized populations. These barriers perpetuate cycles of unintended pregnancies and health inequity, as contraception remains inaccessible to those who need it most.

### Breaking free: method discontinuation as an act of resistance

Discontinuation of contraceptive pills can be seen as a form of resistance against the dominant healthcare norms and systemic pressure experienced by the former pill users we interviewed. The interviewed gynecologists acknowledged that many patients discontinued contraceptive pills to explore “natural rhythms” or “reduce side effects”. Some also suggested observing one’s body afterward. Former users stopping the pill because of side effects or exploring their bodies without hormones described a critical shift. Cumulative biopsychological harm from pill use, seen as an adverse reaction and detachment from natural cycles, is intolerable. By discontinuing, these individuals resist systemic pressures that prioritize hormonal contraception and downplay their physical and emotional impact.

Fannie: I thought: ‘I have had enough at some point (laughing). I could take a break or put it down for good. […] And I was not feeling well in general, but not anything specific, but this feeling was that something was not right. I just had no connection at all with my period, what it is, and how it works, and I thought maybe I have hormonal problems or whatever and if I want kids one day, I do not want to find out that day that I have problems with it. (laughing). So, yeah, I kind of thought; this is time.

This reclamation of bodily awareness without artificial hormones rejects the pathologization of natural biological processes and portrayal of the female body as needing medical control.

For participants, resistance against the cumulative impact of contraceptive pills signifies a movement towards personal autonomy and challenges the normalization of and systemic focus on contraceptive responsibility, hormonal contraception, and a biomedicalized framing of reality. In their resistance to the dominance of the pill, former users advocate for transparency, respect for patient autonomy, and equitable health care practices that value individuals’ experiences and choices.

## Discussion

By applying a continuum of symbolic, structural, and slow violence to the empirical case of the contraceptive pill in Germany, we identified the mechanisms through which reproductive autonomy is eroded systematically. Our findings demonstrate this continuum as a causal chain: symbolic violence legitimizes structural barriers, which, in turn, manifest as the slow violence of cumulative harm. This aligns with the European Parliament’s framing of gynecological violence as a systemic phenomenon rooted in gendered power imbalances and institutional failures, rather than isolated incidents [19] and helps to better understand individual experiences of violence and hurt as expressions of societal configurations of power.

Our primary contribution is an empirical demonstration of how these forms of violence intersect. Symbolic violence manifests through the institutional backing of the pill, which subtly reinforces it as the default, almost “naturalized,” option for contraception, placing the reproductive burden on women [30, 39, 76]. This dominance not only acts socially, but is internalized by themselves. This epistemic dismissal of alternatives and lived experiences is a key source of power. As Stevens et al. [34] argue, labeling women’s beliefs as “misconceptions” reinforces clinical authority by devaluing their experiential knowledge. This creates a profound disconnect between providers and users, a finding that resonates with research across different national contexts [9, 12, 77, 78].

Furthermore, our analysis shows that this power imbalance is actively constructed through communication, as theorized by Littlejohn and Kimport [36]. Our interviews with gynecologists showed that they actively negotiate uncertainty about side effects, shaping and challenging its meaning in clinical practice. For instance, providers acknowledged subjective side effects like feeling “sadder” but framed them as having an “unclear” causal relationship. This discursive strategy minimizes women’s experiences and influences their decisions [36–38], as Lily’s narrative painfully illustrates. When dismissed, patients seek validation elsewhere, often in online spaces where misinformation proliferates [12, 79]. This dynamic creates a feedback loop of invalidation that constitutes a form of slow violence, in which the failure to legitimize patients’ embodied knowledge exposes them to long-term, preventable harm.

A key contribution of this study is the nuanced depiction of gynecologists in our study as agents of symbolic violence and subjects of structural violence. Our findings avoid a simplistic perpetrator-victim dichotomy by showing how providers’ intentions are often undermined by systemic constraints. The internal contradiction navigated by providers in our study who espouse an “individual solution” yet default to the pill for adolescents [78, 80] is not simply individual failure but a direct consequence of structural violence, such as the lack of formal training and adequate remuneration they described. Our data empirically validate the theoretical argument that well-intentioned actors can become complicit in reproducing harm within a system that perpetuates structural violence.

Finally, this study deepens our understanding of contraceptive discontinuation as a response to this continuum of violence. Interestingly, the institutional naturalization of the pill is countered by a form of resistance in which individuals mobilize their own ideals of the “natural” body [81]. Our findings empirically support Inoue et al.‘s [35] critique that biomedical frameworks invalidate women’s lived experiences. By choosing to stop the pill to explore their body’s ‘natural rhythms” and regain a “lost connection with the body,” [55], as articulated by Fannie, the former users in our study are resisting the very dismissal that Inoue et al. [35] identified. Their reasons, which might otherwise be relegated to ambiguous categories like ‘personal reasons’ in clinical studies, are here revealed as acts of “strategic norm negotiation” [57]. This act is an embodied reaction to the power dynamics and communicative dismissal highlighted in the literature [34, 36, 37, 80]. By discontinuing, former users reject their position as passive clinical objects and reiterate their reproductive experiences outside the dominant biomedical frameworks.

Ultimately, our analysis makes the mechanisms of reproductive injustice visible, showing that gynecological violence is not a deviation from care but rather a product of systemic arrangements [19]. Our findings point toward a reimagining of gynecological care that actively reduces power asymmetries and supports genuine freedom of choice, centering equity, dignity, and the recognition of the body as a site of self-determination.

### Strengths and limitations

We pioneered the application of a gynecological violence continuum to empirical data, using a multidisciplinary approach within the research team. By explicitly including the perspectives of both former contraceptive pill users and gynecologists, our analysis moves beyond individual-level explanations to reveal how violence is reproduced through organizational and symbolic factors in the healthcare system. This dual-perspective balances power relations [21] in the research process and allows for a more nuanced understanding of how both groups operate within a historically developed health system that reproduces structural violence. We also acknowledge that applying theory to qualitative research imposes meaning from that theoretical framework onto the research phenomenon.

We aimed to counteract symbolic violence through critical self-reflection, acknowledging that such violence remains embedded in social practices, despite intentions. As violence often lacks clear origins, determining intervention points is challenging. However, given the increasing public attention, this study may help transform the structural conditions. As Nixon (2011) noted, change requires a sustained collective engagement.

This study has several limitations must be acknowledged. First, we had difficulty recruiting gynecologists, and the participating physicians were likely more open to critical perspectives on the pill, which might not be representative of the broader profession. Second, the study’s limited and homogeneous sample of primarily cisgender heterosexual participants from Germany restricts the generalizability of its findings. Finally, by focusing solely on former contraceptive pill users, the study may have introduced a selection bias, as participants with negative experiences could be overrepresented.

## Conclusion

In this article, we showed that the recognition of reproductive control as a fundamental human right stands in stark contrast to the reality of gynecological violence, in which harmful practices during medical care can infringe upon an individual’s bodily autonomy and reproductive choices. The interplay of symbolic and structural violence in this context shows how power operates subtly yet pervasively — not through overt denial of care but through policies, incentives, and norms that reshape medical practice from within. Over time, these mechanisms contribute to a form of slow violence, gradually eroding both the provider’s capacity to deliver patient-centered care and the patient’s right to make informed, empowered decisions about their reproductive health. These findings advocate a transition toward more equitable, inclusive, and responsive contraceptive care models that prioritize patient voices and dismantle systemic harm.

## Data availability

Due to the qualitative nature of this study and the need to protect participants’ privacy and confidentiality, the data were not publicly available. Sharing full transcripts or raw data could compromise the anonymity of the individuals involved.

## Supplementary Information

Below is the link to the electronic supplementary material.

Supplementary Material 1

## Data Availability

Due to the qualitative nature of this study and the need to protect participants’ privacy and confidentiality, the data were not publicly available. Sharing full transcripts or raw data could compromise the anonymity of the individuals involved.

## Footnotes

1 Episodic interviews are qualitative interviews that capture how people make sense of their experiences by combining narrative accounts of specific events with semi-structured questions, thus allowing access to both lived experiences and broader attitudes.

2 Pseudonyms are used throughout the text for confidentiality.
