# Supplementary material for: The violence of reproductive injustice: Reflections on birth control and its medical epistemics
Source: Int J Equity Health. 2025 Dec 8;24:342. doi: 10.1186/s12939-025-02727-5 (PMC12690829; doi:10.1186/s12939-025-02727-5)
Supplement: Supplementary file 1 — Supplementary Material 1 [file 12939_2025_2727_MOESM1_ESM.docx]

**Supplementary Table 1. Semantic and Latent Levels of Analytic Themes: Linking Themes to Forms of Gynecological Violence**

| Analytic Theme | Semantic Level (Surface Meaning) | Latent Level (Underlying Power Dynamics) | Forms of Gynecological violence | Key Illustrative Quotes |
| --- | --- | --- | --- | --- |
| 1. Side effects as normalized harmful trade-offs | **Former users***:* Experience of side effects; Side effects (e.g., mood changes, weight gain) are dismissed as minor or inevitable.  **Gynecologists:** Acknowledge the side effects (e.g. low libido, venous thrombosis) to some extent; While some of their patient’s attribute health issues to it, causality is often unclear. | The medical system pathologizes subjective experiences while silencing patient narratives, framing them as non-medical or psychological. | **Structural violence:** The systemic failure to provide transparent, comprehensive information about side effects perpetuates harm and disempowers users.  **Symbolic violence**: Normalization of side effects marginalizes users. Medical authority overrides lived experience.  **Slow violence**: Cumulative harm from ignored symptoms over time. | *Heather*: “[…] it was always very [concentrated] in the direction of ‘Form a medical point of view, the pill is still the best thing there is.’ […]”  *Dr. Pete*: “A significant proportion report that they feel changed in their soul […]” |
| 2. Contraceptive knowledge as a site of power | **Gynecologists:** Scarcity of studies on male contraception; Absence from specialist training in Germany; Influence of the Pharmaceutical industry on clinical knowledge; | Power is concentrated in medical institutions and pharmaceutical industries, shaping what is considered valid or legitimate knowledge. | **Structural violence**: The systemic neglect of comprehensive contraceptive research and training.  **Symbolic violence**: The prioritization of industry-generated knowledge over independent research and formal training perpetuates symbolic violence, as it normalizes corporate influence in clinical practice.  **Slow violence:** Inadequate training and limited contraceptive options, gradually undermining autonomy and deepening health disparities. | *Dr. Freya*: “It is not an issue in gynecological training[…] you do not have to take a course on it.”  *Dr. Elsa*: “We get our information mainly from pharmaceutical speakers.” |
| 3. The hidden costs of contraceptive care | **Gynecologists:** Counseling is underfunded, time-limited, and often not reimbursed. | Economic disincentives devalue patient education, turning informed consent into a privilege accessible only to those who can pay. Reinforcing provider authority and positioning informed choice as a privilege—shaping care around profit, not autonomy. | **Structural violence**: Systemic undervaluation of relational, educational care.  **Symbolic violence**: Procedures are prioritized over dialogue; time is framed as “extra.”  **Slow violence:** Time-limitations and undervaluation, accumulate silently. | *Dr. Pete*: “The 20-minute appointment is often insufficient. […] I charge a small fee so I can take my time… women respond positively […].” |
| 4. The contraceptive pill as the default: disconnect between lived and medical knowledge | **Former users:** Prioritization of the contraceptive pill throughout the implementation process: Brief consultations, dismissal of concerns and failure to consider individual needs.  **Gynecologists:** On the one hand, they aim to find individual contraceptive solutions. However, they make exceptions when it comes to young patients. They express concerns about teenage pregnancies. There are conflicting views about biomedicalization. | Medical authority overrides patient experience, positioning providers as sole decision-makers.  Patient voices are silenced or minimized, reinforcing hierarchical provider-patient relationships.  Young women are constructed as vulnerable, justifying exclusion from long-acting methods and limiting their autonomy.  Power lies in defining what counts as “safe” or “rational” care, marginalizing non-medical or patient-led approaches. | **Symbolic violence:** The pill is presented as the obvious "best" option, which silences patients' voices and marginalizes non-pharmaceutical alternatives.  **Structural violence:** Systemic barriers, poor training, underfunded care, limited access and cost restrict autonomy, especially for marginalized groups.  **Slow violence*:*** The cumulative harm caused by misinformation, ignored symptoms and delayed care erodes autonomy and deepens health inequities over time. | *Lola*: “The decision was pretty clear […] I got the pill and then the issue was over.”  *Dr. Pete*: “contraception advice can only lead to an individual solution“ and “I can’t imagine being the first person to put anything in there.”  Dr. Henry: “I find caring for a teenage pregnancy more stressful, if that is not wanted, than giving good contraceptive advice.” |
| 5. The hidden costs of misinformation | **Former users:** Receive conflicting or oversimplified information about contraception.  **Gynecologists:** They identify gaps in reproductive and sexual education among their patients and criticize economic barriers, especially for low-income individuals. | The medical establishment controls knowledge, which limits patients’ ability to question or critically engage with their care.  Knowledge is distributed unequally, positioning providers as gatekeepers and patients as passive recipients.  Access to information and care is stratified by income, thereby reinforcing socioeconomic hierarchies within the field of reproductive health. | **Structural violence:** Systemic failures, such as inadequate education and economic barriers, create unequal access to information and care. These failures disproportionately affect marginalized populations.  **Slow violence:** The cumulative harm caused by misinformation, ignored symptoms, delayed care and prolonged exposure to harmful norms erodes reproductive autonomy and deepens health inequities over time. | *Hannah*: “my gynecologist that it is a miracle cure-all […],friend's gynecologist: ‘you should not pump your body full of these hormones.’”  *Dr. Marry*: “ […] you can hardly make up for it [the missing education] because that simply does not achieve anything. […] It just quickly becomes a case of, take the pill, do not forget to take it, then you will not get pregnant.”  *Dr. Freya*: “[…] So, I would like to see free access to it.” |
| 6. Breaking free: Method discontinuation as an act of resistance | **Former users:** Cumulative biopsychological harm and the desire for bodily awareness.  **Gynecologists:** Discontinuation is often due to side effects or a desire for bodily awareness. | Discontinuation is a way of resisting cumulative bodily harm, hormones and biomedical control. | **Structural violence:** It is an act of resistance against systemic barriers, such as lack of access to alternatives, poor education and dismissal by providers, that deny real choice.  **Symbolic violence**: It challenges the normalised belief that the pill is the 'natural' or 'best' choice, resisting biomedical control over women's bodies.  **Slow violence**: Resistance emerges after prolonged harm. | *Fannie*: “I thought: ‘I have had enough […]. I just had no connection at all with my period’” |
